# Association Between Maternal Serum Folate Concentrations in the First Trimester and the Risk of Birth Defects: The Hokkaido Study of Environment and Children’s Health

**DOI:** 10.2188/jea.JE20170185

**Published:** 2019-04-05

**Authors:** Kumiko Ito, Tomoyuki Hanaoka, Naomi Tamura, Seiko Sasaki, Chihiro Miyashita, Atsuko Araki, Sachiko Ito, Hisanori Minakami, Kazutoshi Cho, Toshiaki Endo, Tsuyoshi Baba, Toshinobu Miyamoto, Kazuo Sengoku, Akiko Tamakoshi, Reiko Kishi

**Affiliations:** 1 Department of Public Health, Hokkaido University Graduate School of Medicine, Sapporo, Japan; 2 Hokkaido University Center for Environmental and Health Sciences, Sapporo, Japan; 3 Department of Nursing, Faculty of Health Science, Hokkaido University of Science, Sapporo, Japan; 4 Department of Health Sciences, Hokkaido University Graduate School of Medicine, Sapporo, Japan; 5 Department of Obstetrics and Gynecology, Hokkaido University Graduate School of Medicine, Sapporo, Japan; 6 Department of Obstetrics and Gynecology, School of Medicine, Sapporo Medical University, Sapporo, Japan; 7 Department of Obstetrics and Gynecology, Asahikawa Medical University, Asahikawa, Hokkaido, Japan

**Keywords:** serum folate concentrations, folic acid, birth defects, pregnancy, first trimester

## Abstract

**Background:**

Low red blood cell folate concentrations during early pregnancy might cause neural tube defects. However, the association between folate concentrations and birth defects of other neural crest cell-derived organs remains unknown. We investigated the associations between birth defects and first-trimester serum folate concentrations in a birth-cohort study in Japan.

**Methods:**

In total, 14,896 women who were prior to 13 weeks of gestation were enrolled from 2003 through 2012. Birth defect information was obtained from medical records and questionnaires. The association between folate levels in the first trimester and birth defects categorized as ICD-10 cord defects and neural crest cell-derived organ defects was examined. The crude and adjusted odds ratios (ORs) and 95% confidence intervals (CIs) per log-transformed folate concentration were calculated using logistic regression.

**Results:**

Blood samples were obtained at a mean of 10.8 weeks of gestation. Median serum folate level was 16.5 (interquartile range, 13.4–21.5) nmol/L, and the deficiency level (less than 6.8 nmol/L) was 0.7%. There were 358 infants with birth defects. The adjusted odds ratio for any birth defect, ventricular septal defects, and cleft lip was 0.99 (95% CI, 0.74–1.32), 0.63 (95% CI, 0.30–1.33), and 4.10 (95% CI, 0.96–17.58), respectively. There were no significant associations between first-trimester maternal serum folate and the risk of birth defects.

**Conclusions:**

We were unable to demonstrate a relationship between maternal serum folate in the first trimester and birth defects. Potential confounding factors may have influenced our results.

## INTRODUCTION

Birth defects cause infant deaths and decrease the quality of life of affected children, adults, and their families, making them an important public health issue.^1^^,^^2^ Although the proportion of birth defects caused by multifactorial inheritance is higher than that caused by a specific factor, such as a chromosomal or genetic defect, the etiology of most birth defects remains unknown.

Folic acid supplementation at preconception and during the first trimester reduces the risk of delivering an infant with neural tube defects (NTDs).^3^^–^^5^ Therefore, in 1998, the United States, Canada, and Costa Rica mandated folic acid fortification of food, with reported declines in NTDs (19–46%) beginning 3–6 years after initiating the fortification.^6^ The neural tube develops from neural crest cells (NCCs). These cells are also involved in the development of the heart and face. The relationship between these congenital malformations and folic acid has been studied; however, the association between folate intake and congenital heart defects (CHDs)^7^^–^^10^ and orofacial clefts^11^^–^^14^ (defects that develop from the same NCCs as the neural tube) remains unknown. Controversy arises in part because of differences in disease definition and type, dose, and intake time of folic acid supplements.^11^

Most studies evaluating the association between maternal folate intake and birth defects were case-control studies,^7^^–^^10^^,^^14^^,^^15^ and data on folate intake during pregnancy was usually obtained retrospectively from the mother after birth, resulting in potential recall bias. In addition, in vivo activity of ingested folic acid differs, because of genetic polymorphisms related to folic acid metabolism.^16^ In some studies, postpartum serum or red blood cell (RBC) folate concentrations were measured in women whose infants had CHDs or orofacial clefts^17^^–^^22^; however, postpartum folate level measurements cannot be used to evaluate the role of folate in organogenesis. In one prospective cohort study examining the association between serum folate during pregnancy and birth defects, serum folate in mid-pregnancy (15^th^–18^th^ gestational weeks) was not associated with conotruncal heart defects or cleft lip with/without cleft palate (CL ± P).^12^^,^^23^ In another prospective study, the first trimester mean serum folate level was significantly lower in mothers of infants with congenital malformations than in mothers of infants without these malformations,^24^ thus, the results are inconsistent. Many studies have examined the relationship between periconceptional folic acid intake and birth defects in Europe, the United States, and China.^25^^,^^26^ In Japan, there is no legislation requiring grain fortification with folic acid, and the Ministry of Health and Welfare only officially recommended in 2000 that women planning to conceive take supplements with 400 µg folic acid daily from the month before conception through the first 3 months after conception to reduce the risk of NTDs.^27^ However, the supplement intake rate of pregnant Japanese women is low, about 30%.^28^

The Hokkaido Study on Environmental and Children’s Health (Hokkaido Study) is a prospective cohort study that has been carried out in the Hokkaido Prefecture of Japan since 2003. Our previous studies reported the incidence of birth defects^29^ and the influence of serum folate concentrations and smoking status in early pregnancy on birth weight.^30^

In this study, we investigated the association between first-trimester maternal serum folate levels and the risk of birth defects by using data from the Hokkaido Study.

## METHODS

### Study cohort and population

**Figure 1.**
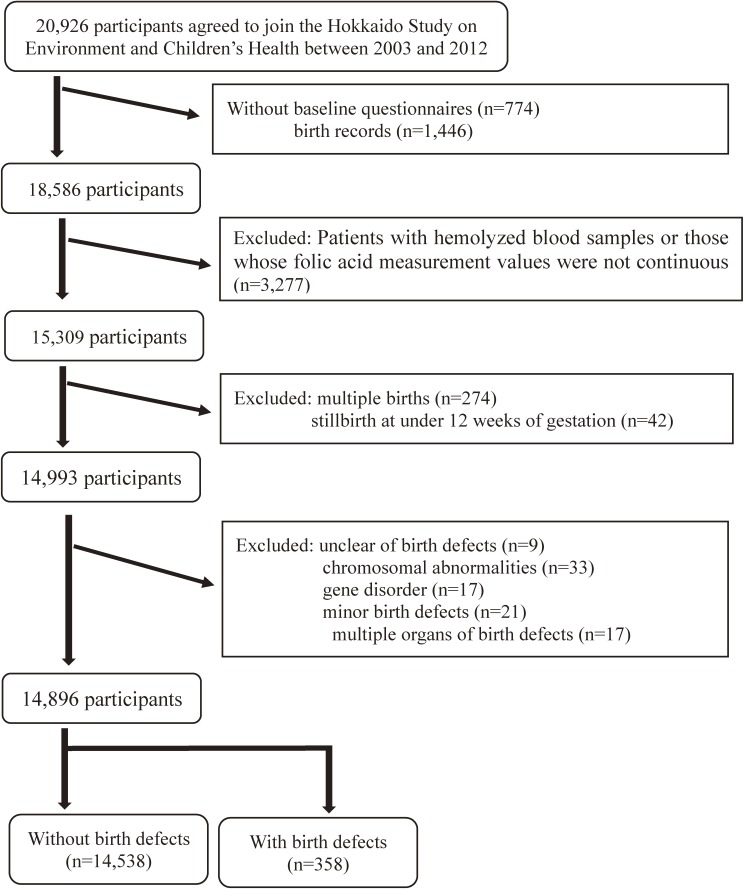
Flowchart of inclusion and exclusion criteria for the study population

The Hokkaido Study investigated the effects of perinatal environmental chemical exposure on birth outcomes, including birth defects, development, and allergies. We enrolled women during early pregnancy (<13 weeks gestational age) visiting one of the 37 gynecological hospitals and clinics within Hokkaido, the northernmost prefecture in Japan. The study design was previously described in detail.^31^^–^^33^ Data were gathered using baseline questionnaires, biochemical assays, birth records, and 1- and 2-year postpartum questionnaires. Figure 1 shows the flow diagram of study participants. A total of 20,926 expectant mothers were initially enrolled from February 2003 through March 2012. We acquired the self-administered questionnaires completed during early pregnancy, the mothers’ medical records at delivery, and the first-trimester serum folate levels. We excluded 3,277 subjects whose serum folate concentrations could not be measured quantitatively because of hemolysis and those whose measurement results were not continuous values. After applying all exclusion criteria and excluding 274 for multiple births and 42 for miscarriages at <12 weeks of gestation, 14,993 mothers were included. To investigate the environmental effect of folic acid on organogenesis, we excluded infants with birth defects of known etiology, such as chromosomal abnormalities; a known gene disorder; only minor anomalies (such as an accessory tag, umbilical hernia, or ear canal fistula); or two or more unrelated birth defects affecting at least two different organ systems. Finally, we analyzed data for 14,896 mother-infant pairs. We obtained written informed consent for all subjects. The institutional ethics boards of the Hokkaido University Center for Environmental and Health Sciences (March 22, 2012) and Hokkaido University Graduate School of Medicine (May 31, 2003) approved the study protocol.

### Data collection

Data were collected from baseline self-administered questionnaires, hospital birth records, and 1- and 2-year postpartum self-administered questionnaires. Participants completed questionnaires during early pregnancy; information obtained included maternal age, parity, maternal educational level, socioeconomic status, medical and reproductive histories, preconception maternal body mass index (BMI; kg/m^2^), use of assisted reproductive technology, family history of birth defects, alcohol consumption, smoking habits, and medication use (prescription or over-the-counter drugs). We defined maternal alcohol and tobacco use as any reported maternal alcohol and tobacco use before pregnancy and medication use as any reported maternal medication use during the first 12 weeks of gestation.

Data regarding all peripregnancy supplements were collected, including supplement type, frequency, and timing. We defined folate supplement users as mothers who consumed one of the following supplements before or during the first 12 weeks of pregnancy: supplements with a main ingredient of folic acid and multivitamins containing folic acid. Within 7 days after the end of the pregnancy, pregnancy outcomes were collected, including the type of birth (miscarriage, stillbirth, and live birth [singleton or multiple]), gestational age, infant sex, birth weight, maternal medical history during pregnancy, obstetric events, and birth defects identified by the delivery unit physicians. Additionally, we collected self-administered questionnaires from all mothers at 1 and 2 years postpartum to obtain additional information including birth defects.

Non-fasting serum blood samples were obtained to measure serum folate concentrations during the first trimester. Serum folate levels were assayed at a commercial laboratory (SRL, Inc. Tokyo, Japan) using an automated competitive protein binding chemiluminescent enzyme immunoassay with the ADVIA Centaur technique (sensitivity, 0.91 nmol/L; coefficient of variation, 4.0–4.3%; imprecision, <10.0%).^31^

### Definition and classification of birth defects

We obtained birth defect information from medical records at birth and self-reported questionnaires at one and two years postpartum, because some diseases, such as CHDs and cryptorchidism, may be diagnosed even after one year following birth. The birth defect was selected from a list of 55 disease names on medical records by physicians, or if the disease was not on the list, described disease names in the unified sheet. These 55 birth defects listed on the unified sheet are possible effect markers of environmental exposure.^29^ We coded the birth defects according to the International Statistical Classification of Disease and Related Health Problems 10th revision (ICD-10).^34^ Further, we defined a group of birth defects resulting from the defective development of NCCs. The neural crest is the fold of neural ectoderm at the junction between the neural and epidermal ectoderm in neurula-stage vertebrate embryos. NCCs give rise to a number of cell types and to a number of tissues and organs, such as the heart, craniofacial skeleton, connective tissues, and smooth muscles.^35^ NTDs are birth defects in the neural tube originating from NCCs, and there has been confirmation that folic acid supplementation prevents the first trimester occurrence of NTDs.^16^ In order to investigate the preventive effect of folic acid on birth defects of other organs developing from NCCs, we considered NTDs,^3^^–^^5^ CHDs,^7^^,^^9^ orofacial clefts,^13^^,^^14^ hypospadias,^36^ and limb reduction defects^37^ as birth defects of organs arising from NCCs.

CHDs were classified into five subgroups: 1) isolated septal defects, including isolated ventricular septal defects and isolated atrial septal defects; 2) conotruncal heart defects, including transposition of the great arteries, tetralogy of Fallot, truncus arteriosus, and double-outlet right ventricle; 3) left-sided obstructive malformations, including aortic valve stenosis, hypoplastic left heart syndrome and its variants, coarctation aorta, and interrupted aortic arch; 4) right-sided obstructive malformations, including pulmonary valve stenosis, pulmonary atresia, tricuspid atresia, and Ebstein anomaly, and 5) other CHDs, including complex heart defects, single ventricle, and isolated persistent ductus arteriosus.^8^^–^^10^ The classification of reported birth defects was confirmed by a neonatal specialist.

### Statistical analysis

Serum folate concentrations showed skewed distribution; therefore, folate data were log transformed (natural log) before analysis. Serum folate concentrations were expressed as the median (interquartile range [IQR]), and other continuous variables were expressed as the mean (standard deviation [SD]), while categorical variables were expressed as percentages.

When comparing data between mothers of infants with birth defects and mothers of infants without birth defects, the Mann-Whitney U-test was used as a non-parametric test for serum folate concentrations. We calculated crude and adjusted odds ratios (ORs) and 95% confidence intervals (CIs) per log-transformed folate concentration in maternal serum folate to establish the association between serum folate concentrations and birth defects using logistic regression. The analyses were adjusted for the following potential confounding factors: maternal age at baseline (continuous variable), parity (0, ≥1), education level (≤12 years, >12 years), maternal BMI at preconception (continuous variable), assisted reproductive technologies (yes, no), and use of alcohol (yes, no), cigarettes (yes, no), and medications (yes, no) during early pregnancy. Two-tailed *P* values <0.05 and 95% CIs that excluded 1.0 were considered statistically significant. We analyzed the data using SPSS for Windows, version 21.0 (IBM Corp., Armonk, NY, USA).

## RESULTS

Subject characteristics at baseline are shown in Table 1. The mean maternal age was 29.8 (SD, 4.8) years. The number of folic acid supplement users was 3,315 (22.6%). Among all mothers, 14,538 (97.6%) had infants without birth defects, while 358 (2.4%) had infants with birth defects. There were 106 (0.7%) miscarriages, 26 (0.2%) induced abortions (under 22 weeks of gestation), 46 (0.3%) stillbirths (after 22 weeks of gestation), and 14,715 (98.8%) live births (3 unknown). Mean gestational age was 38.6 (SD, 2.7) weeks. Mean venous blood sampling time for serum folate measurement were taken at 10.8 (SD, 1.7) weeks of pregnancy. The maternal median serum folate level was 16.5 (IQR, 13.4–21.5) nmol/L, and low serum folate (less than 6.8 nmol/L, according to WHO criteria^38^) was only 0.7%. Notably, median serum folate concentration of all mothers, whether or not their infants were born with birth defects, was 16.5 nmol/L. The serum folate concentrations of sub-groups and phenotypes are shown in Table 2 and Table 3.

**Table 1.** Baseline characteristics of participants (*n* = 14,896)

Characteristics	*n* (%)		Characteristics	*n* (%)	
Maternal age, years			Smoking^a^		
Mean (SD)	29.8	(4.8)	Yes	5,793	(39.3)
<20	177	(1.2)	No	8,929	(60.7)
20–24	1944	(13.1)	Unknown	174	—
25–29	4969	(33.4)	Assisted reproductive technology		
30–34	5209	(35.0)	Yes	592	(4.0)
≥35	2596	(17.4)	No	14,206	(95.4)
Unknown	1	—	Unknown	98	—
Prepregnancy body mass index, kg/m^2^			Diabetes mellitus		
Mean (SD)	21.2	(3.4)	Yes	66	(0.5)
<18.5	2,525	(17.3)	No	14,037	(99.5)
18.5–24.9	10,420	(71.6)	Unknown	793	—
≥25.0	1,615	(11.1)	Family history of birth defects		
Unknown	336	—	Yes	322	(2.2)
Education level, years			No	14,457	(97.8)
≤12	7,302	(49.4)	Unknown	117	—
>12	7,480	(50.6)	Medication use^b^		
Unknown	114	—	Yes	5,577	(37.7)
Parity, times			No	9,201	(62.3)
0	5,963	(42.5)	Unknown	118	—
≥1	8,066	(57.5)	Any folic acid supplement^c^		
Unknown	867	—	(including multivitamin)		
Alcohol^a^			Yes	3,315	(22.6)
Yes	1,951	(13.2)	No	11,321	(77.4)
No	12,793	(86.9)	Unknown	260	—
Unknown	152	—			

SD, standard deviation.

^a^Maternal alcohol use and smoking were considered for mothers who had reported these before pregnancy.

^b^Maternal medication use was considered for mothers who had reported any medication use until 12 weeks of pregnancy.

^c^Total group of folic acid supplements comprised of folic acid alone and folic acid-containing multivitamins.

**Table 2.** Associations between natural log-transformed maternal serum folate concentration during the first trimester and any birth defects and ICD-10 code birth defects after 12 weeks of gestation compared with mothers of infants without birth defects

	Number of infants with birth defects	Maternal serum folate (nmol/L)	Crude ORs (95% CIs)	Adjusted ORs (95% CIs)
Any birth defect	358	16.5 (13.4, 22.0)	1.04 (0.79–1.36)	0.99 (0.74–1.32)
ICD-10 code birth defects				
Nervous system, Q00-07	12	16.9 (14.9, 25.8)	1.68 (0.42–6.78)	1.35 (0.28–6.48)
Eye, ear, face, and neck, Q10-18	12	16.0 (14.3, 20.3)	0.87 (0.20–3.77)	1.33 (0.29–6.10)
Circulatory system, Q20-28	130	16.5 (13.4, 22.2)	1.09 (0.70–1.69)	0.99 (0.61–1.61)
Respiratory system, Q30-34	1	16.5 (—)	0.04 (0.00–5.69)	0.03 (0.00–3.58)
Cleft lip and cleft palate, Q35-37	24	18.3 (15.3, 21.9)	1.56 (0.58–4.21)	1.94 (0.66–5.80)
Digestive system, Q38-45	18	18.1 (13.4, 21.5)	2.18 (0.71–6.68)	3.03 (0.90–10.21)
Genital organs, Q50-56	63	16.3 (12.9, 21.7)	0.97 (0.52–1.83)	0.91 (0.45–1.83)
Urinary system, Q60-64	25	15.9 (13.6, 18.8)	0.55 (0.19–1.56)	0.47 (0.15–1.45)
Musculoskeletal system, Q65-79	61	16.3 (12.5, 20.4)	0.77 (0.40–1.48)	0.61 (0.30–1.24)
Other Skin, Q80-85 (except for syndromes, not elsewhere classified)	12	19.3 (12.5, 28.1)	1.57 (0.39–6.38)	2.18 (0.50–9.52)

CI, confidence interval; OR, odds ratio.

Serum folate, median (interquartile range). Serum folate level of women who had infants without birth defects was 16.5 (13.4, 21.5) nmol/L.

ORs (with 95% CIs) per log-transformed folate concentration in maternal serum folate level.

Adjusted for maternal age (continuous variable), parity, educational level (years), assisted reproductive technology, smoking, alcohol, body mass index (continuous variable), and medication use.

**Table 3.** Associations between natural log-transformed maternal serum folate concentration during the first trimester and neural crest cell-derived birth defects after 12 weeks of gestation compared with mothers of infants without birth defects

	Number of infants with birth defects	Maternal serum folate (nmol/L)	Crude ORs (95% CIs)	Adjusted ORs (95% CIs)
All birth NCC defects	161	16.5 (13.5, 21.7)	1.09 (0.73–1.62)	1.02 (0.65–1.58)
NTDs, Q00,01,05	6	19.9 (14.9, 26.5)	2.52 (0.37–17.25)	2.01 (0.21–19.05)
CHDs, Q20–28	125	16.5 (13.4, 20.6)	0.94 (0.60–1.48)	0.83 (0.50–1.37)
CHDs classifications				
Septal heart defects, Q21.0–21.1	80	16.4 (13.3, 21.0)	1.04 (0.59–1.82)	0.91 (0.49–1.70)
Conotruncal heart defects, Q20.0, 20.1, 20.3, 21.3	5	16.5 (15.9, 20.6)	1.01 (0.11–9.49)	0.74 (0.08–7.19)
Left ventricle outflow obstruction, Q23.0, 23.3–4, 25.1–3	9	14.9 (12.9, 16.5)	0.30 (0.05–1.76)	0.23 (0.03–1.68)
Right ventricle outflow obstruction Q21.3, 22.1, 22.5, 25. 6	17	16.1 (13.8, 26.3)	1.11 (0.33–3.71)	0.91 (0.25–3.27)
CHDs phenotype				
Ventricular septal defects, Q21.0	61	16.3 (12.7, 19.9)	0.73 (0.38–1.40)	0.63 (0.30–1.33)
Atrial septal defects, Q21.1	12	22.2 (13.7, 30.5)	3.30 (0.86–12.62)	2.27 (0.49–10.53)
Pulmonary valve stenosis, Q22.1	17	16.1 (13.8, 26.3)	1.19 (0.36–3.95)	0.93 (0.26–3.31)
Orofacial cleft phenotype				
Cleft lip with or without cleft palate, Q36–37	13	19.0 (15.9, 21.3)	2.25 (0.60–8.38)	4.10 (0.96–17.58)
Cleft palate (alone), Q35	8	18.9 (13.9, 22.7)	0.81 (0.13–4.87)	0.47 (0.06–3.62)
Hypospadias, Q54	4	21.6 (12.1, 35.1)	2.86 (0.28–29.65)	1.86 (0.10–34.66)
Limb reduction, Q71–72	2	16.8 (15.9, 17.7)	0.83 (0.02–30.19)	2.92 (0.05–157.08)

CHDs, congenital heart defects; CI, confidence interval; NCCs, neural crest cells; NTDs, neural tube defects; OR, odds ratio.

Serum folate, median (interquartile range). Serum folate level of women who had infants without birth defects was 16.5 nmol/L.

ORs (with 95% CIs) per log-transformed folate concentration in maternal serum folate.

Adjusted for maternal age (continuous variable), parity, educational level (years), assisted reproductive technology, smoking, alcohol, body mass index (continuous variable), and medication use.

CHDs were classified by a previous study.^8^^–^^10^

Circulatory system defects were the most frequent birth defects (130 infants), followed by genital organ defects (63 infants) (Table 2). Ventricular septal defects (61 infants) were the most common within a phenotype. Table 2 shows the ORs of having an infant with birth defects based on maternal folate concentrations. As log-transformed maternal serum folate concentration increased one unit, the adjusted ORs of birth defects of the urinary system (Q60–64) and musculoskeletal system (Q65–79) decreased; however, this was not statistically significant (OR 0.47; 95% CI, 0.15–1.45 and OR 0.61; 95% CI, 0.30–1.24, respectively). On the other hand, the adjusted ORs of the nervous system (Q00–07) and cleft lip and cleft palate (Q35–37) increased; however, this was also not statistically significant (1.35 [95% CIs, 0.28–6.48], 1.94 [95% CIs, 0.66–5.80], respectively). Moreover, maternal serum folate levels in the first trimester were not significantly associated with the risk of other birth defects.

We estimated the association between maternal serum folate levels in the first trimester and the risk of birth defects from NCCs (Table 3). As log-transformed maternal serum folate concentration increased one unit, the adjusted ORs of all CHDs and ventricular septal defects decreased (OR 0.83; 95% CI, 0.50–1.37 and OR 0.63; 95% CI, 0.30–1.33, respectively). The adjusted ORs for atrial septal defects and cleft lip were 2.27 (95% CI, 0.49–10.53) and 4.10 (95% CI, 0.96–17.58), respectively.

## DISCUSSION

This prospective study investigated the association between first-trimester serum folate and birth defects. Elevated maternal serum folate concentrations increased the adjusted ORs for birth defects of the nervous system, cleft lip and cleft palate, while the adjusted ORs for birth defects of the urinary and musculoskeletal systems decreased. In birth defects from NCCs, elevated maternal serum folate concentrations increased the adjusted ORs for atrial septal defect and cleft lip, and reduced the adjusted ORs for ventricular septal defects. However, there were no significant associations found between serum folate levels and any birth defects. The NTD risk increased with higher serum folate concentrations; however, because of the small number of NTDs, this result may have been due to chance.

Some prospective studies have investigated the association between folate levels and risk of CHDs and CL ± P. A study in Saudi Arabia^24^ reported that the mean first-trimester serum folate levels of mothers of infants with congenital malformations (40.85 nmol/L) were significantly lower than those in mothers of infants without these malformations (50.50 nmol/L; *P* < 0.001), unlike the results of our study. However, their study had few subjects, and potential confounding factors such as socioeconomic status, prepregnancy BMI, and smoking habits were not mentioned.^24^ These factors may have influenced the results. In California, Shaw et al^14^^,^^23^ estimated the association between birth defects and serum folate levels at 15–18 gestational weeks by comparing the folate levels of mothers of infants with only conotruncal heart defects or CL ± P to mothers of infants without birth defects. They observed no association between serum folate levels and conotruncal heart defects or CL ± P risk, in agreement with results of the present study. Moreover, the Shaw study ascribed the lack of association between serum folate levels and conotruncal heart defects or CL ± P to the fact that their participants were from a population whose food was fortified with folic acid. The United States began mandatory folic acid fortification in 1998, and the prevalence of low serum folate levels (less than 3.0 ng/mL or 6.8 nmol/L) among women of childbearing age was approximately 0.8% from 1999–2006.^39^

In previous studies on serum folate concentrations in pregnant women during the first trimester in Japan, Takimoto^40^ and Matsuzaki^41^ reported that the median serum folate level was 23.2 nmol/L (51 pregnant women) and 4.8 ng/mL (10.9 nmol/L, value converted by the author, 118 pregnant women), respectively. Serum folate concentration of our participants approximated the middle value of the serum folate values of the two aforementioned studies. Most of our participants were also mothers with serum folate levels within the normal range (13.5–45.3 nmol/L) per WHO criteria,^38^ which might help to explain the lack of a significant association between these levels and birth defects.

Folate is integral to one-carbon metabolism, which produces pyrimidines and purines for the synthesis of DNA and S-adenosylmethionine. Accordingly, folic acid is essential for cell proliferation and/or cell survival.^42^ Folic acid may affect cell proliferation in the early stages of development, thereby promoting posterior neural tube closure.^43^ The association between folic acid supplementation or serum (or RBC) folate levels and birth defects other than NTDs from NCCs has been inconsistent. However, folate status is also affected by gene polymorphisms related to folate metabolism,^16^ and gene-environment interactions between gene polymorphisms related to folate metabolism with periconceptional folate supplementation have been observed for CHDs and cleft lip.^44^^–^^46^ Furthermore, folate deficiency may modulate the risk of congenital malformations by affecting the bioavailability of methyl groups for DNA methylation reactions or nucleotide synthesis. However, we could not explain the role of folic acid in organogenesis. In a large-scale study such as a national population-based study, a multi-center hospital-based case-control design,^3^^,^^9^^,^^10^^,^^13^ folic acid supplementation has been shown to reduce the risk of CHD or cleft lip. On the other hand, one study reported that the risk of cleft lip increased with the intake of folic acid supplements before and after conception.^14^ To determine any adverse effects of the intake of folic acid on the fetus, further research is required, and careful consideration is necessary.

The strengths of our study are its birth cohort study design and our inclusion of first-trimester evaluations. Most previous studies of birth defects have used folate intake as a proxy for folate during pregnancy. To elucidate the role of folic acid during the organogenesis period, we measured serum folate during the critical period of organ development. In addition, information on possible confounding factors such as smoking, drinking, and medications taken in early pregnancy allowed us to analyze the effect of lifestyle on the risk of folate deficiency without recall bias. Second, our study enrolled women attending community hospitals and clinics as well as university hospitals. Therefore, the frequency of birth defects in this study likely reflects that of the general population of Hokkaido Prefecture.^29^

This study also has several limitations. First, we obtained birth defect information from medical records at birth and supplemented it with self-reported maternal information from questionnaires completed 1 to 2 years after delivery. Therefore, birth defect phenotypes could have been misclassified because they were not medically diagnosed. Second, non-fasting serum folate levels were treated as indicators of folate status. In general, RBC folate concentrations have been viewed as a better long-term measure of folate concentrations. However, the determination of RBC folate is more complex, and serum folate measurement is more common than erythrocyte folate measurement in medical facilities in Japan. Serum folate assays are preferred in large epidemiological studies; therefore, we used serum folate evaluation. We sampled non-fasting blood. With folate-containing food, peak concentrations of plasma folate occur in a few hours, returning to pre-ingestion concentrations at around 8 hours.^47^ In our study, blood samples were collected without the recording of elapsed time after meals. Thus, there is a possibility that the concentration of folate in the obtained serum may have been higher than it would have been in a fasting blood sampling. However, this consequence should have occurred in all mothers, whether or not they had children with birth defects. Third, although the cohort was relatively large, there were few birth defects. In our previous study, the spina bifida incidence was 1.6 per 10,000 births.^29^ For spina bifida, the minimum required sample size is approximately 130,000 when two dependent variables are included in the logistic regression analysis.^48^ Finally, potential confounding variables, including environmental factors such as exposure to radiation or air pollution, and infectious diseases, gene polymorphisms, and gene-interactions, were not assessed in this study. Not considering these factors might have resulted in the finding of a non-significant relationship between maternal serum folate levels in the first trimester and birth defects.

In this study, we investigated the association between maternal serum folate levels in the first trimester and birth defects; however, we were unable to establish a significant association between them. In future studies, we will examine the association between folate levels, gene polymorphisms of folic acid metabolic enzymes, and birth defects using a nested case-control design or case-cohort design.
